# Co-expression Network Analysis of Biomarkers for Adrenocortical Carcinoma

**DOI:** 10.3389/fgene.2018.00328

**Published:** 2018-08-15

**Authors:** Lushun Yuan, Guofeng Qian, Liang Chen, Chin-Lee Wu, Han C. Dan, Yu Xiao, Xinghuan Wang

**Affiliations:** ^1^Department of Urology, Zhongnan Hospital of Wuhan University, Wuhan, China; ^2^Department of Endocrinology, The First Affiliated Hospital of Zhejiang University, Hangzhou, China; ^3^Department of Urology, Massachusetts General Hospital, Harvard Medical School, Boston, MA, United States; ^4^Greenebaum Cancer Center, School of Medicine, University of Maryland, Baltimore, MD, United States; ^5^Laboratory of Precision Medicine, Zhongnan Hospital of Wuhan University, Wuhan, China; ^6^Department of Biological Repositories, Zhongnan Hospital of Wuhan University, Wuhan, China

**Keywords:** adrenocortical carcinoma (ACC), weighted gene co-expression network analysis (WGCNA), cell cycle, biomarker, progression and prognosis

## Abstract

Adrenocortical carcinoma (ACC) is a rare malignancy with a poor prognosis. And currently, there are no specific diagnostic biomarkers for ACC. In our study, we aimed to screen biomarkers for disease diagnosis, progression and prognosis. We firstly used the microarray data from public database Gene Expression Omnibus database to construct a weighted gene co-expression network, and then to identify gene modules associated with clinical features of ACC. Though this algorithm, a significant module with *R*^2^ = 0.64 (*P* = 9 × 10^-5^) was identified. Co-expression network and protein–protein interaction network were performed for screen the candidate hub genes. Checked by The Cancer Genome Atlas (TCGA) database, another independent dataset GSE19750, and GEPIA database, using one-way ANOVA, Pearson’s correlation, survival analysis, diagnostic capacity (ROC curve) and expression level revalidation, a total 12 real hub genes were identified. Gene ontology and KEGG pathway analysis of genes in the significant module revealed that the hub genes are significantly enriched in cell cycle regulation. Moreover, gene set enrichment analysis suggests that the samples with highly expressed hub genes are correlated with cell cycle. Taken together, our integrated analysis has identified 12 hub genes that are associated with the progression and prognosis of ACC; these hub genes might lead to poor outcomes by regulating the cell cycle.

## Introduction

Adrenocortical carcinoma is a rare malignancy. Its incidence is approximately 0.5–2 new cases per million people per year (Fassnacht et al., 2011), with an increased occurrence during childhood and the fourth to fifth decades of life. Most ACCs occur sporadically, but some cases are associated with various genetic diseases, e.g., Li-Fraumeni syndrome (LFS), Beckwith–Wiedemann, and multiple endocrine neoplasia type I (MEN1). ACCs are often aggressive, cannot be completely resected and have a poor prognosis (Fassnacht et al., 2011; Else et al., 2014). In a study of 330 ACCs from M. D. Anderson, the median survival was 3.2 years and median overall survival for stage I, II, III, and IV were 24.1, 6.08, 3.47, and 0.89 years, respectively (Ayala-Ramirez et al., 2013). The 275 surgically resected tumors had a median local-recurrence free time of 1 year. In addition, a study of 3982 ACCs from 1985 to 2005 in United States exhibited that the survival did not improve (Beuschlein et al., 2015). Based on these facts, it is urgent to investigate the mechanisms that promote ACC progression and to discover novel molecular biomarkers for the prognosis.

With the development of high-throughput microarray technology, many studies have reported the genes involving in tumor progression (Chen L. et al., 2017; Robertson et al., 2017). Gene expression profiles have been used to identify genes associated with progression of ACCs in some previous studies (Pinto et al., 2015; Kulshrestha et al., 2016). However, these studies were mostly concerned with differentially expressed genes and did not consider the high degree of interconnection between genes, where genes with similar expression patterns may be functionally related. Merely screening the differentially expressed genes in normal and tumor samples had limitations and we should pay more attention to correlation between gene expression and clinical features. The algorithm, WGCNA can construct free-scale gene co-expression networks to explore the relationships between different gene sets or between gene sets and clinical features (Langfelder and Horvath, 2008). Currently, WGCNA is used to study various cancers and has identified biomarkers associated with critical features. However, it was not used in ACC samples and our group was the first to use this algorithm in ACC. Thus, we attempt to construct a co-expression network of relationships between genes through a systematic biology approach based on a weighted genome expression network and to identify network-centric genes associated with clinical features of ACC and then use various datasets and databases (GEO, TCGA, GEPIA, and STRING) to validate the value of the hub genes.

## Materials and Methods

### Data Collection

Expression profiles of mRNA and related clinical data of human ACCs were downloaded from Gene Expression Omnibus (GEO) database^1^. Dataset GSE10927 (Giordano et al., 2009) performed on Affymetrix Human Genome U133 Plus 2.0 Array was used as a training set to construct co-expression networks and identify hub genes in this study. This dataset included human samples of 33 ACCs, 22 adrenocortical adenomas, and 10 normal adrenal cortex samples, each from a different patient. The additional independent dataset GSE19750 (Demeure et al., 2013) based on the same platform as the training set was downloaded from GEO and used as a test set to verify our results. This dataset included 44 ACC tissues and four normal adrenal glands. Moreover, RNA-sequencing data of 79 ACC samples were also downloaded from The Cancer Genome Atlas (TCGA) database^2^ to further verify our results. The gene expression data were based on the RNA-sequencing technology IlluminaHiseq.

### Data Preprocessing

**FIGURE 1:**
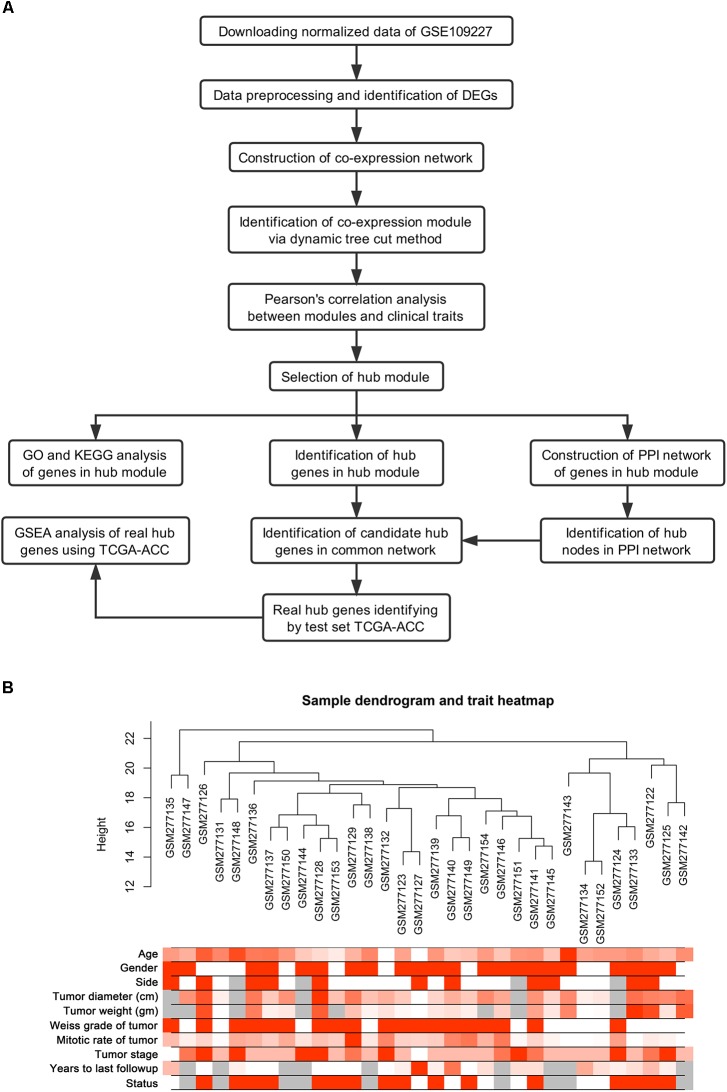
Study design and clustering dendrogram of 32 tumor samples as well as the clinical traits. **(A)** Flow diagram of data preparation, processing, analysis, and validation in this study. **(B)** The clustering was based on the expression data of differentially expressed genes between tumor samples and normal samples in ACC. The color intensity was proportional to older age, gender, side, tumor diameter (cm), tumor weight (gm), weiss grade of tumor, mitotic rate of tumor, tumor stage, years to last follow-up and status. Color: Female: white, Male: red; Status: live: white, dead: red.

Normalized data were first downloaded from GEO database. Microarray quality was assessed by sample clustering according to the distance between different samples in Pearson’s correlation matrices (Gautier et al., 2004). A brief study design is shown in **Figure 1A**.

### Differentially Expressed Genes (DEGs) Screening

The “limma” (linear models for microarray data) (Ritchie et al., 2015) R package was used to screen the DEGs between normal adrenal gland and ACC. The SAM (significance analysis of microarrays) with FDR < 0.05 and | log2 FC| > 0.263 were chosen as the cut-off criteria to select genes further considered in the network construction (Feng et al., 2016; Ling et al., 2017; Ma et al., 2017; Wang C.F. et al., 2017; Xiong et al., 2017; Yan et al., 2017; Yang et al., 2017). The differentially expressed genes between normal adrenal cortex and ACC in two dataset GSE10927 and dataset GSE19750 were screened.

### Co-expression Network Construction

First, DEG expression data profiles were evaluated to determine whether the samples and genes were of sufficient quality. Then, we used the “WGCNA” package in R to construct scale-free co-expression network for the DEGs. To ensure that the results of network construction were reliable, outlier samples (which were distant from other samples in the clustering via average linkage method) were removed. An appropriate soft threshold power was selected in accordance with standard scale-free networks, with which adjacencies between all differential genes were calculated by a power function (Clarke et al., 2013; Botia et al., 2017). Then, the adjacency was transformed into a TOM, and the corresponding dissimilarity (1-TOM) was calculated. Module identification was accomplished with the dynamic tree cut method by hierarchically clustering genes using 1-TOM as the distance measure with a deepSplit value of two and a minimum size cutoff of 50 for the resulting dendrogram. To further analyze the module, we calculated the dissimilarity of module eigengenes (MEs), chose a cut line for module dendrogram and merged some modules.

### Identification of Clinical Significant Modules

Two approaches were used to identify modules related to clinical traits of ACC. First, gene significance (GS) was defined as the log10 transformation of the *P* value (GS = lgP) in the linear regression between gene expression and the clinical traits. In addition, module significance (MS) was defined as the average GS for all the genes in a module. In general, the module with the absolute MS ranked first or second among all the selected modules was considered as the one related to a clinical trait. MEs were considered as the major component in the principal component analysis for each gene module and the expression patterns of all genes could be summarized into a single characteristic expression profile within a given module. In addition, we calculated the correlation between MEs and clinical traits to identify the relevant module. The module with the maximal absolute MS among all the selected modules was usually considered to be related to a clinical trait. Finally, the module highly correlated with certain clinical traits was selected for further analysis.

### Identification of Candidate Hub Genes

Hub genes in the co-expression network were defined by module connectivity, measured by absolute value of the Pearson’s correlation and clinical trait relationship. We identified hub genes in module that were highly correlated with certain clinical traits. Furthermore, we uploaded all genes in the hub module to the Search Tool for the Retrieval of Interacting Genes (STRING) database^3^ (Szklarczyk et al., 2015, 2017), choosing a confidence > 0.4 to construct a PPI. In the PPI network, genes with a connectivity degree ≥ 4 (node/edge) were defined hub nodes. Hub genes common to both the co-expression network and PPI network were regarded as candidate hub genes for further analysis.

### Real Hub Genes Identification

In the GSE19750 test set, linear regression analyses and survival analysis were performed to validate the role of hub genes in the progression and prognosis of ACC. Moreover, we used the Gene Expression Profiling Interactive Analysis (GEPIA) database^4^ (Tang et al., 2017) to perform survival analysis and stage plots of the candidate hub genes. The ANOVA test was performed using GSE19750 and TCGA-ACC data. Meanwhile, ROC curve analysis was performed and the AUC was calculated to distinguish the tumor tissues from the normal tissues as well as the malignant tumors from the benign tumors by using training set GSE10927 and test set GSE19750. Genes with a significant *P* value in survival analyses, linear regression analyses, stage plots, common DEGs in GSE19750 and GSE10927, ANOVA test and AUC ≥ 0.85 were defined as the “real” hub genes. Then the real hub genes were performed the Pearson’s correlation between their expression levels and MKi67 by using GSE10927, GSE19750, and TCGA-ACC.

### Functional and Pathway Enrichment Analysis

The Database for Annotation, Visualization and Integrate Discovery^5^ (Dennis et al., 2003; Sherman et al., 2007) is an online program providing a comprehensive set of functional annotation tools for investigators to understand biological meaning behind large list of genes. Enriched biological themes of DEGs in hub modules, particularly GO terms, were identified, and those on KEGG pathway maps were visualized using DAVID. We chose the top 10 terms including the candidate hub genes to be the key BP and pathways. *P* < 0.05 was set as the cut-off criterion.

### Gene Set Enrichment Analysis (GSEA)

In the TCGA-ACC data, 79 ACC samples were divided into two groups according to the median expression level of hub genes. To identify potential function of the hub gene, GSEA^6^ (Subramanian et al., 2005) was conducted to detect whether a series of *a priori* defined BPs were enriched in the gene rank derived from DEGs between the two groups.

For use with GSEA software, the collection of annotated gene sets of c2.cp.kegg.v6.0.symbols.gmt in Molecular Signatures Database (MSigDB)^7^ was chosen as the reference gene set. ES ≥ 0.6 and FDR < 0.05 were chosen as the cut-off criteria. We chose the terms enriched in all hub genes as the potential function of the hub genes.

## Results

### DEG Screening

After data preprocessing and quality assessment, the expression matrices were obtained from the 65 samples in training set GSE10927. Under the threshold of FDR < 0.05 and | log_2_ FC| > 0.263, a total of 1956 DEGs (666 up-regulated and 1290 down-regulated in ACC samples) were selected for subsequent analysis (**Supplementary Table S1**). In the test set GSE19750, a total of 3810 DEGs (1263 up-regulated and 2547 in ACC samples) were identified (**Supplementary Table S2**).

### Weighted Co-expression Network Construction and Key Modules Identification

**FIGURE 2:**
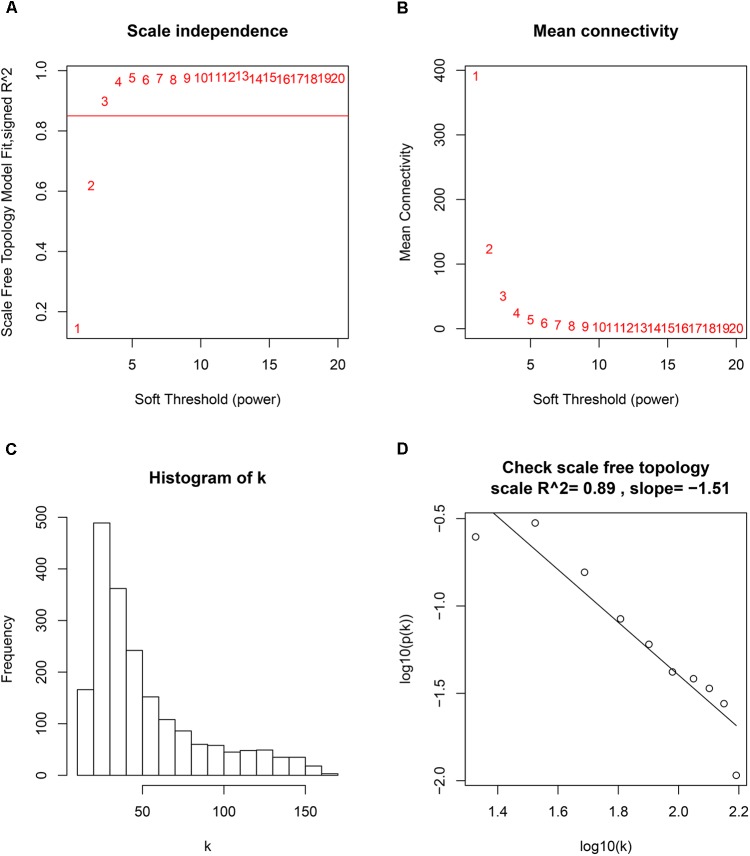
Determination of soft-thresholding power in the weighted gene co-expression network analysis (WGCNA). **(A)** Analysis of the scale-free fit index for various soft-thresholding powers (β). **(B)** Analysis of the mean connectivity for various soft-thresholding powers. **(C)** Histogram of connectivity distribution when β = 3. **(D)** Checking the scale free topology when β = 3.

**FIGURE 3:**
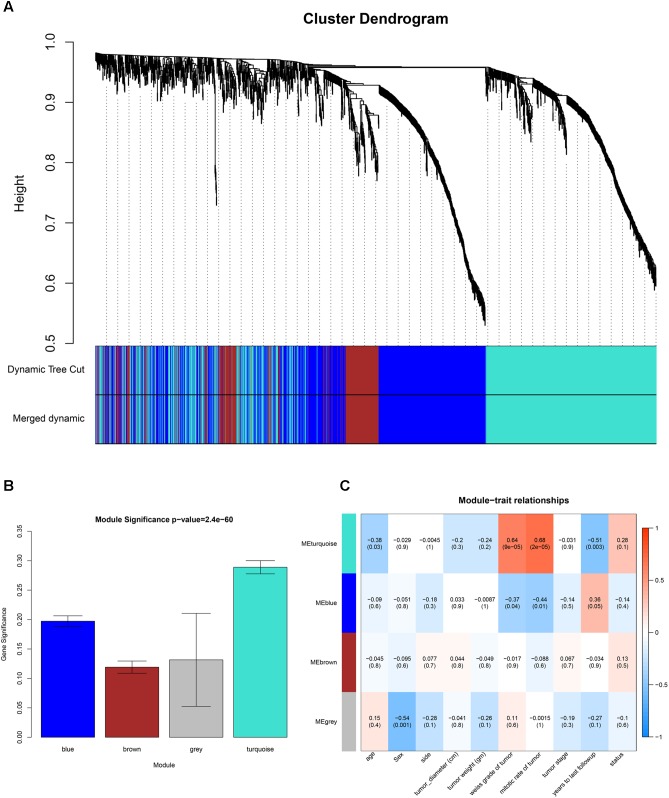
Identification of modules associated with clinical information. **(A)** Dendrogram of all differentially expressed genes clustered based on a dissimilarity measure (1-TOM). **(B)** Heatmap of the correlation between module eigengenes (MEs) and different clinical information of ACC (age, gender, side, tumor diameter (cm), tumor weight (gm), weiss grade of tumor, mitotic rate of tumor, tumor stage, years to last follow-up and status). **(C)** Distribution of average gene significances and errors in the modules associated with the weiss grade of ACC.

GSM277130 was an outlier sample and was removed from subsequent analysis in GSE10927 (**Supplementary Figure S1**). A total of 33 samples with clinical data were included in the co-expression analysis (**Figure 1B**). Using the “WGCNA” package in R, DEGs with similar expression patterns were grouped into modules via the average linkage hierarchical clustering. In this study, the power of β = 3 (scale free *R*^2^ = 0.89) was selected as the soft-thresholding to ensure a scale-free network (**Figure 2**). A total of four modules were identified (**Figure 3A**). Two methods were used to test the relevance between each module and the ACC clinical information; here, we focused on the tumor grade. First, modules with a greater MS were considered to have more connection with tumor grade. However, most of the correlations were low to moderate (*R*^2^ < 0.5), and we found that the MS of the turquoise module (*P* = 9 × 10^-5^, *R*^2^ = 0.64) was higher than that of any other module (**Figure 3B**). Afterward, the ME of the turquoise module showed a higher correlation with tumor grade than did other modules (**Figure 3C**). Based on the two methods, the turquoise module with tumor grade was identified as the clinical significant module, which was extracted for further analysis.

### Identification of Candidate Hub Genes for Tumor Grade

**FIGURE 4:**
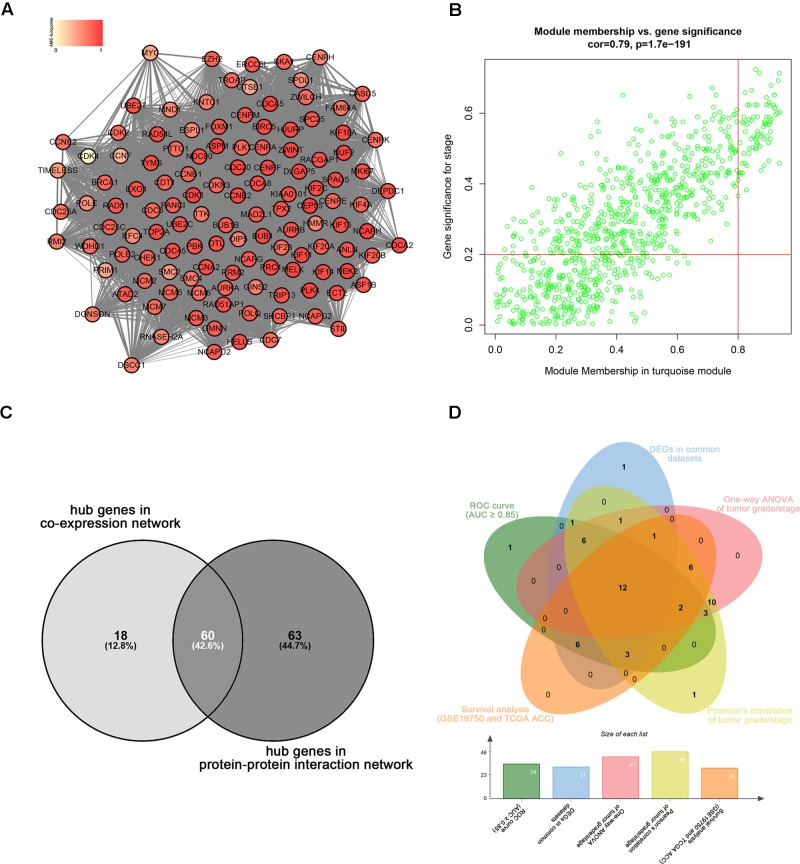
Hub genes detection. **(A)** Protein–protein interaction network of the hub nodes. **(B)** Scatter plot of MEs in turquoise module. **(C)** Common hub genes in the co-expression network and PPI network. **(C)** Common genes with significant p value in survival analysis, ROC curve analysis, one-way ANOVA, Pearson’s correlation analysis and DEGs analysis.

Highly connected hub genes in a module play important roles in the BPs. As to the PPI network, under the cutoff of confidence > 0.4 and connectivity degree of ≥ 4 (node/edge), 123 genes were identified as hub genes (**Figure 4A**) and the whole PPI network were showed in **Supplementary Figure S2**. Defined by module connectivity and measured by absolute value of the Pearson’s correlation and clinical trait relationship, 78 genes with high connectivity in the turquoise module were taken as candidate hub genes in the co-expression network (**Figure 4B**). A total of 60 genes were identified both in PPI network and co-expression network identified as candidate hub genes (**Figure 4C**).

### Real Hub Gene Identification

**FIGURE 5:**
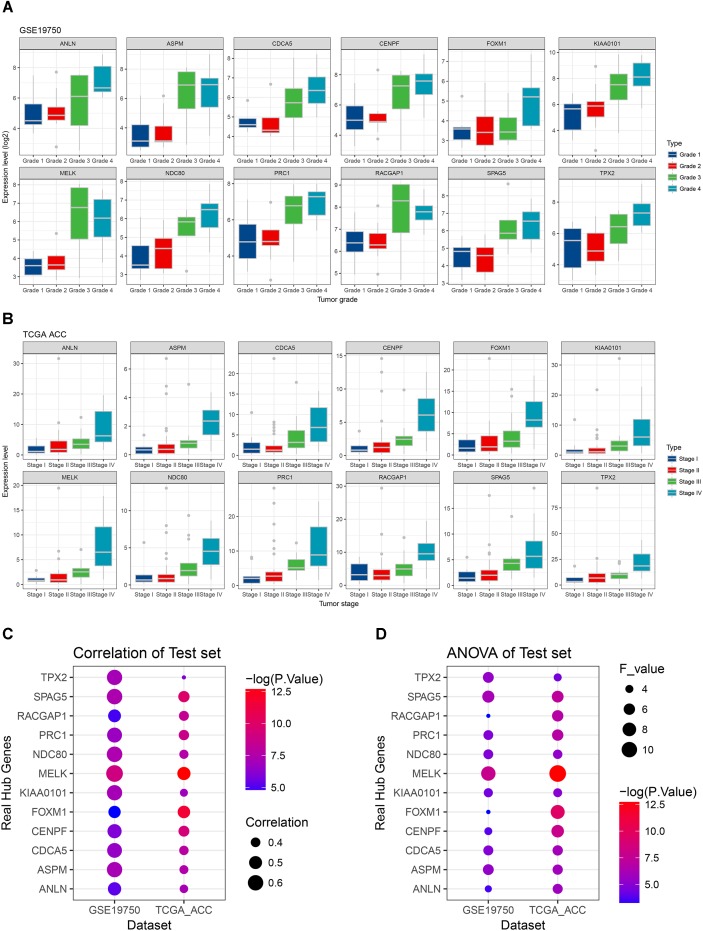
Stage and grade plot of the real hub genes. **(A)** Grade plot of the real hub genes using test set GSE19750. **(B)** Stage plot of the real hub genes using test set TCGA-ACC. **(C)** Pearson’s correlation analysis of the real hub genes using test set GSE19750 and TCGA-ACC. **(D)** One-way ANOVA analysis of the real hub genes using test set GSE19750 and TCGA-ACC.

**FIGURE 6:**
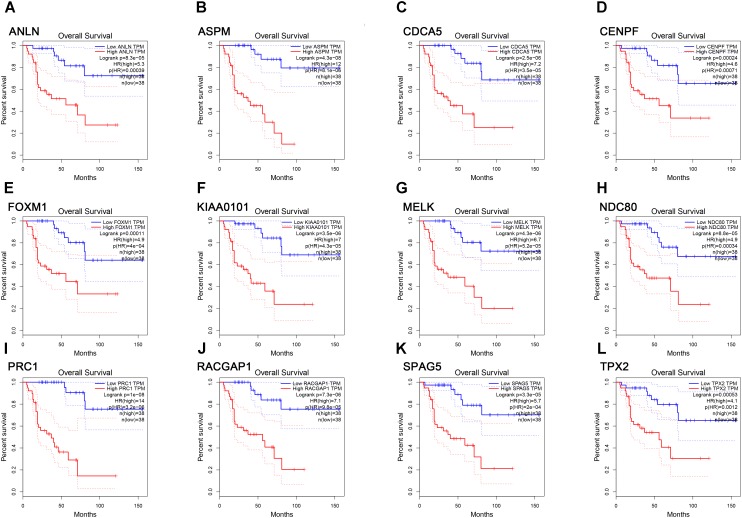
Survival analysis of the association between the expression levels of real hub genes and overall survival time in ACC (based on TCGA data in GEPIA). **(A)** *ANLN*, **(B)** *ASPM*, **(C)** *CCNA5*, **(D)** *CENPF*, **(E)** *FOXM1*, **(F)** *KIAA0101*, **(G)** *MELK*, **(H)** *NDC80*, **(I)** *PRC1*, **(J)** *RACGAP1*, **(K)** *SPAG5*, **(L)** *TPX2*. Red line indicates the samples with gene highly expressed, and blue line shows the samples with gene lowly expressed. HR, hazard ratio.

**FIGURE 7:**
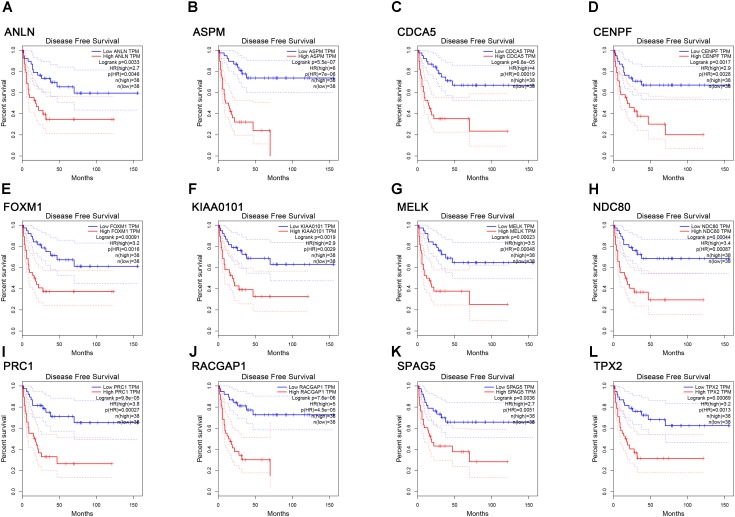
Survival analysis of the association between the expression levels of real hub genes and disease free survival time in ACC (based on TCGA data in GEPIA). **(A)** *ANLN*, **(B)** *ASPM*, **(C)** *CCNA5*, **(D)** *CENPF*, **(E)** *FOXM1*, **(F)** *KIAA0101*, **(G)** *MELK*, **(H)** *NDC80*, **(I)** *PRC1*, **(J)** *RACGAP1*, **(K)** *SPAG5*, **(L)** *TPX2*. Red line indicates the samples with gene highly expressed, and blue line shows the samples with gene lowly expressed. HR, hazard ratio.

**FIGURE 8:**
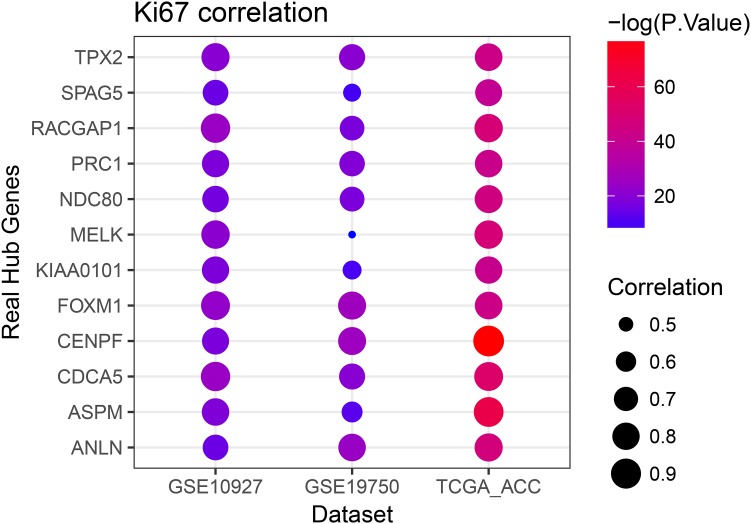
Pearson’s correlation analysis of the association between the Ki67 expression and the expression of real hub genes.

Linear regression analyses and one-way ANOVA analysis were conducted to validate candidate hub genes in the test set GSE19750 and TCGA-ACC (**Supplementary Table S3**), and totally 46 and 41 genes were identified, respectively. As tumor progression always affects tumor prognosis, we validated the real hub genes by investigating their roles in ACC prognosis using GSE19750 and TCGA-ACC (**Supplementary Table S4**) and 30 genes showed significant *P* value in both test sets. To further test the diagnostic capacity of the candidate hub genes, ROC curve was performed and the AUC were calculated (**Supplementary Table S5**), and 34 genes were identified with AUC ≥ 0.85. In addition, we also investigated the expression levels of candidate hub genes in two datasets (GSE19750 and GSE10927), and 31 were differentially expressed in both datasets (GSE19750 and GSE10927). Common genes with significant *P* value in those five analyses were screened as the real hub genes, and 12 genes were eventually identified (ANLN, ASPM, CDCA5, CENPF, FOXM1, KIAA0101, MELK, NDC80, PRC1, RACGAP1, SPAG5, and TPX2) (**Figure 4D**). The grade or stage plot, Pearson’s correlation, ANOVA test results and survival analyses of the real hub genes were showed in **Figures 5**–**7** and **Supplementary Figures S3**–**S5**. MKi67 is a biomarker for tumor proliferation, the Pearson’s correlation of MKi67 and real hub genes were performed (**Figure 8**).

### Functional and Pathway Enrichment Analysis

**FIGURE 9:**
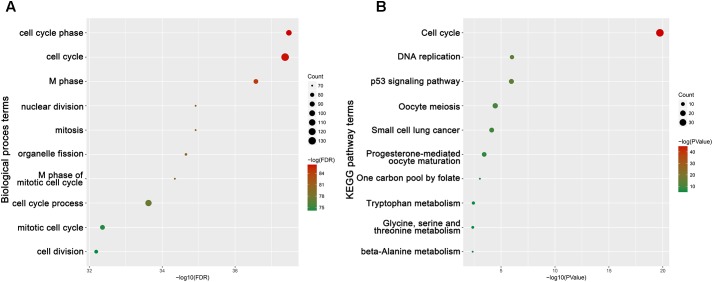
Bioinformatics analysis of genes in turquoise module. **(A)** GO analysis and **(B)** KEGG pathway enrichment of hub genes.

To obtain further insight into the function of DEGs in hub module, the DEGs were uploaded to the DAVID database. GO analysis results showed that hub genes were enriched in the top 10 BPs, including cell cycle phase, cell cycle, M phase, nuclear division, mitosis, organelle fission, M phase of mitotic cell cycle, cell cycle process, mitotic cell cycle and cell division. Moreover, hub genes were quite significantly enriched in cell cycle (**Figure 9**).

### Gene Set Enrichment Analysis

To identify the potential function of those hub genes in ACC, GSEA was conducted to search BPs enriched in any hub gene highly expressed samples. Seven gene sets were enriched in the samples with all hub genes highly expressed, including “base excision repair,” “cell cycle,” “DNA replication,” “mismatch repair,” “nucleotide excision repair,” “spliceosome,” and “homologous recombination” (**Supplementary Figure S6**).

## Discussion

Adrenocortical carcinoma is one of the most aggressive solid tumors in humans, with an overall poor prognosis (Fassnacht et al., 2011; Else et al., 2014). Successful surgical resection for early stage disease is the only curative treatment for ACC. However, the risk of recurrence is high. Therefore, it is particularly important to stratify patients with ACC into low- or high-risk groups to monitor disease recurrence and assign them to appropriate therapeutic interventions. Recently, a large multicentric study by the European Network for the Study of Adrenal Tumors has demonstrated that the Ki67 labeling index (Li) is the most powerful parameter predicting disease recurrence and survival in ACC patents after complete tumor resection (Beuschlein et al., 2015). Recent studies have attached values to biomarkers for outcomes of ACC; however, carcinogenesis does not occur due to a few altered genes. Thus, it is necessary to identify novel molecular biomarkers that can predict disease stage and clinical outcome of ACC patients; these biomarkers could play critical roles in pathogenesis and enable personalized treatment.

Weighted genes co-expression network analysis was used to construct gene co-expression networks on the basis of similarities of expression profiles among samples and then illustrate a global interpretation of genes. Many articles have been published using WGCNA to screen clinical-feature related biomarkers in several types of tumors (Chen P. et al., 2017; Wang F. et al., 2017; Yuan et al., 2017). This study is the first to use the WGCNA algorithm for the purpose of identifying potential biomarkers for ACC. Based on the clinical features (age, gender, tumor side, tumor diameter, tumor weight, Weiss grade of tumor, mitotic rate of tumor, tumor stage, years to last follow, and living status), we eventually screened the gene co-expression modules related to the tumor grade of ACC. The turquoise module was identified, and 78 hub genes were derived from the module in co-expression network; meanwhile, 123 hub genes were identified in PPI analysis. Relating the results of PPI network, 60 hub nodes were screened in both the co-expression module and PPI network. Further regression analysis, survival analysis, one-way ANOVA and ROC curve analysis were performed, and 12 real hub genes were finally selected that were associated with poor outcomes and tumor stage of ACC. Interestingly, we found that all real hub genes differed greatly between tumor, begin or normal tissue in the test set and GEPIA database. Additionally, among the 12 real hub genes, only few biomarkers were identified in previous studies. Jain et al. (2011) discovered that *KIAA0101*, which is a marker of cellular proliferation and promotes growth and invasion, was a good diagnostic marker for distinguishing benign from malignant adrenocortical neoplasm. Although other hub genes were not reported to participate in ACC progression, they were reported to be involved in various tumors. TPX2, overexpressed in cancers, is being established as marker for the diagnosis and prognosis of malignancies, and the functions of TPX2 are attributed to its Ran-regulated microtubule-associated protein properties and to its control of the Aurora A kinase (Neumayer et al., 2014). SPAG5 is reported as a poor prognostic biomarker in cervix cancer, breast cancer and bladder cancer (Yuan et al., 2014; Abdel-Fatah et al., 2016; Liu et al., 2018). RACGAP1 as a proliferation marker was indicated to involve in carcinogenesis of many cancers (Ke et al., 2013; Milde-Langosch et al., 2013; Imaoka et al., 2015; Bornschein et al., 2016; Sahin et al., 2016). In recent study, researchers found that abnormal PRC1 expression correlates with poor patient outcome in various malignancies, which may be caused by PRC1-mediated CIN and aneuploidy (Li et al., 2018). NDC80 was reported to associate with poor outcomes and tumor proliferation in multiple cancers via cell cycle regulation (Qu et al., 2014; Meng et al., 2015; Xing et al., 2016). In previous studies, MELK functioned as a modulator of intracellular signaling and affects various cellular and BPs, including cell cycle, cell proliferation, apoptosis, spliceosome assembly, gene expression, embryonic development, hematopoiesis, and oncogenesis (Jiang and Zhang, 2013). FOXM1 transcription factor is a regulator of myriad BPs, including cell proliferation, cell cycle progression, cell differentiation, DNA damage repair, tissue homeostasis, angiogenesis and apoptosis; meanwhile, elevated FOXM1 expression was found in most cancers, suggesting it has an integral role in tumorigenesis and recent research findings also placed FOXM1 at the center of cancer progression and drug sensitivity (Koo et al., 2012). Interestingly, co-expression of FOXM1 and CENPF is a robust prognostic indicator of poor survival and metastasis (Aytes et al., 2014). CDCA5 was identified as a negative prognostic marker in bladder cancer, breast cancer, gastric cancer and hepatocellular carcinoma (Chang et al., 2015; Phan et al., 2018; Shen et al., 2018; Zhang et al., 2018). ASPM played an important role in pancreatic tumor and glioma progression, in vascular invasion, early recurrence and poor prognosis of hepatocellular carcinoma and even represented a potential target for radiotherapy (Lin et al., 2008; Bikeye et al., 2010; Kato et al., 2011; Wang et al., 2013). According to the previous studies, we found all the hub genes participated in the tumorigenesis.

Enrichment analyses for the turquoise module indicated that BPs focused on cell cycle regulation, including cell cycle phase, cell cycle, M phase, nuclear division, mitosis, organelle fission, M phase of mitotic cell cycle, cell cycle process, mitotic cell cycle and cell division. Meanwhile, KEGG enrichment analysis revealed that genes in the turquoise module were significantly enriched in cell cycle. To further study the mechanism by which these genes regulate tumorigenesis, we performed GSEA analysis using ACC RNA Seq data from TCGA. All hub genes were enriched in base excision repair, cell cycle, DNA replication, homologous recombination, mismatch repair, nucleotide excision repair and spliceosome. The two enrichment analyses demonstrated that hub genes were closely related to the function of cell cycle regulation. Previous studies demonstrated that the presence of p53 was necessary for DNA-damaged cells to arrest, repair the damage, and reenter the cell cycle (Lukin et al., 2015). Cell cycle is a continuous and accurate process; meanwhile, monitoring and regulating the cell cycle are important (Xu, 2006). Deregulation of cell cycle is closely related to carcinogenesis and tumor progression (Kaistha et al., 2016). Regarding our candidate hub genes that were enriched in the function of regulating cell cycle, we hypothesize that they might influence ACC by affecting cell cycle.

## Conclusion

In conclusion, we used weighted gene co-expression analysis to construct a gene co-expression network for ACC for the first time, as well as identified and validated network hub genes associated with tumor grade. Interestingly, candidate hub genes showed significant prognostic value. Meanwhile, we predicted the potential function of the stage- and prognosis-related hub genes, which participated in cell cycle regulation. These hub genes had potential roles in the translational medicine and might become therapeutic targets. However, our study only selected potential biomarkers of ACC that were associated with tumor progression and prognosis, and further molecular biological experiments are needed to confirm the function of candidate biomarkers in ACC.

## Author Contributions

LY, YX, and XW conceived and designed the study. LY, GQ, LC, and YX performed the analysis procedures. LY, GQ, C-LW, HD, and YX analyzed the results. LY, GQ, and XW contributed analysis tools. LY, GQ, and YX contributed to the writing of the manuscript. All authors reviewed the manuscript.

## Conflict of Interest Statement

The authors declare that the research was conducted in the absence of any commercial or financial relationships that could be construed as a potential conflict of interest.

## Footnotes

1 http://www.ncbi.nlm.nih.gov/geo/

2 https://genome-cancer.ucsc.edu/

3 http://www.string-db.org/

4 http://gepia.cancer-pku.cn/

5 http://david.abcc.ncifcrf.gov/

6 http://software.broadinstitute.org/gsea/index.jsp

7 http://software.broadinstitute.org/gsea/msigdb/index.jsp

## Abbreviations

ACC: adrenocortical carcinoma
BP: biological process
DAVID: the database for annotation, visualization, and integrated discovery
FC: fold change
FDR: false discovery rate
GO: gene ontology
GSEA: gene set enrichment analysis
PPI: protein–protein interaction
TOM: topological overlap matrix
WGCNA: weighted gene co-expression network analysis.
