# Supplementary material for: Co-expression Network Analysis of Biomarkers for Adrenocortical Carcinoma
Source: Front Genet. 2018 Aug 15;9:328. doi: 10.3389/fgene.2018.00328 (PMC6104177; doi:10.3389/fgene.2018.00328)
Supplement: Supplementary file 6 [file Image_1.PDF]

Supplementary Figure S1

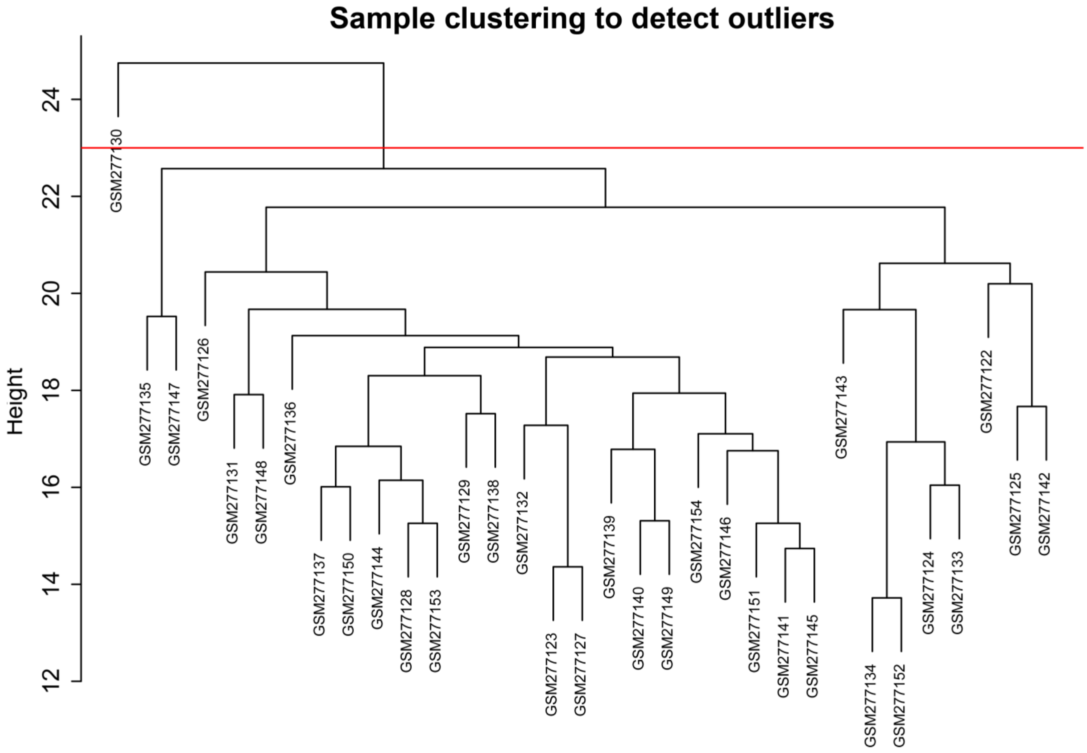

Supplementary Figure S1. Sample clustering to detect outliers (training set GSE10927).
