# Supplementary material for: Co-expression Network Analysis of Biomarkers for Adrenocortical Carcinoma
Source: Front Genet. 2018 Aug 15;9:328. doi: 10.3389/fgene.2018.00328 (PMC6104177; doi:10.3389/fgene.2018.00328)
Supplement: Supplementary file 7 [file Image_2.PDF]

Supplementary Figure S2

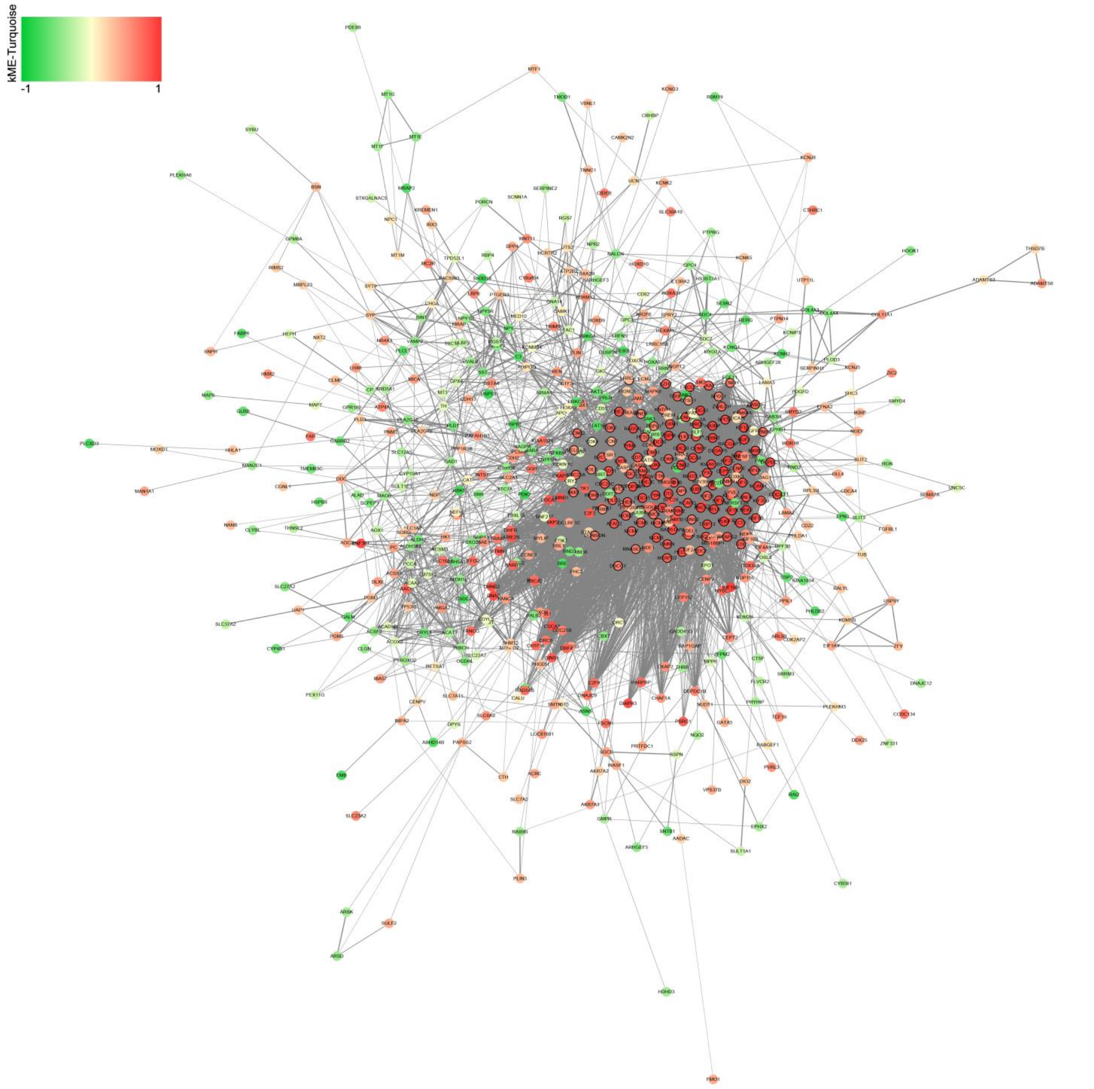

Supplementary Figure S2. Protein-protein interaction network of the genes in hub module. Protein-protein interaction network of genes in the turquoise module. The color intensity of each node was proportional to the correlation with clinical traits (positive correlation in red and negative correlation in green). The nodes with bold circle represented network hub genes in PPI network. The edge width was proportional to the score of protein-protein interaction based on the STRING database.
