# Supplementary material for: Co-expression Network Analysis of Biomarkers for Adrenocortical Carcinoma
Source: Front Genet. 2018 Aug 15;9:328. doi: 10.3389/fgene.2018.00328 (PMC6104177; doi:10.3389/fgene.2018.00328)
Supplement: Supplementary file 8 [file Image_3.PDF]

Supplementary Figure S3

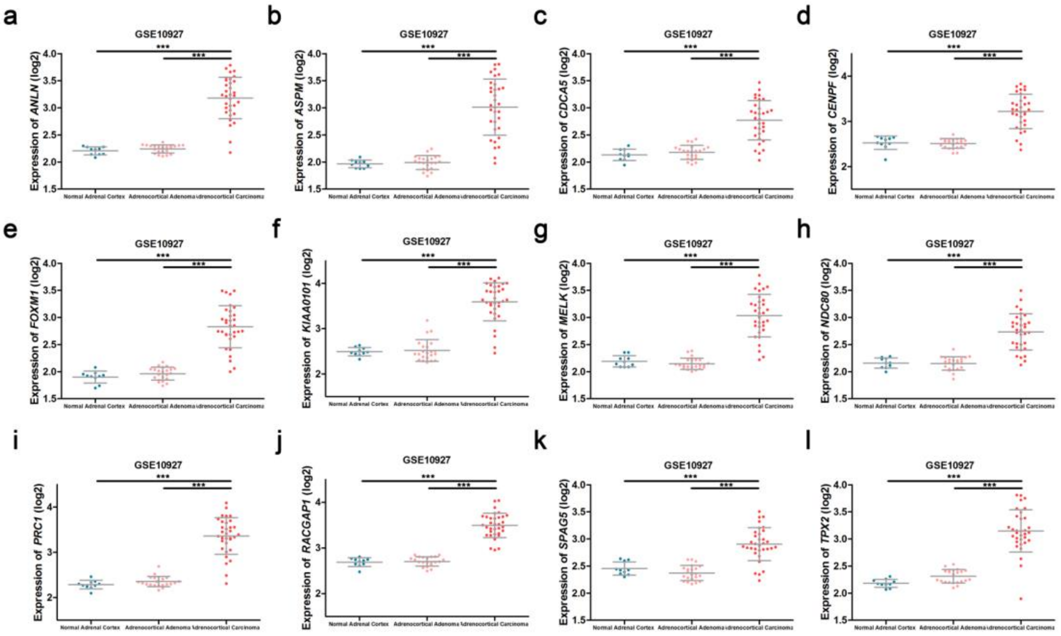

Supplementary Figure S3. Validation of the real hub genes using GSE10927. Validation of the gene expression levels of real hub genes between normal adrenal cortex, adrenocortical adenoma and adrenocortical carcinoma samples (based on microarray data of GSE10927). (a) ANLN, (b) ASPM, (c) CDCA5, (d) CENPF, (e) FOXM1, (f) KIAA0101, (g) MELK, (h) NDC80, (i) PRC1, (j) RACGAP1, (k) SPAG5, (l) TPX2.
