# Supplementary material for: Co-expression Network Analysis of Biomarkers for Adrenocortical Carcinoma
Source: Front Genet. 2018 Aug 15;9:328. doi: 10.3389/fgene.2018.00328 (PMC6104177; doi:10.3389/fgene.2018.00328)
Supplement: Supplementary file 9 [file Image_4.PDF]

Supplementary Figure S4

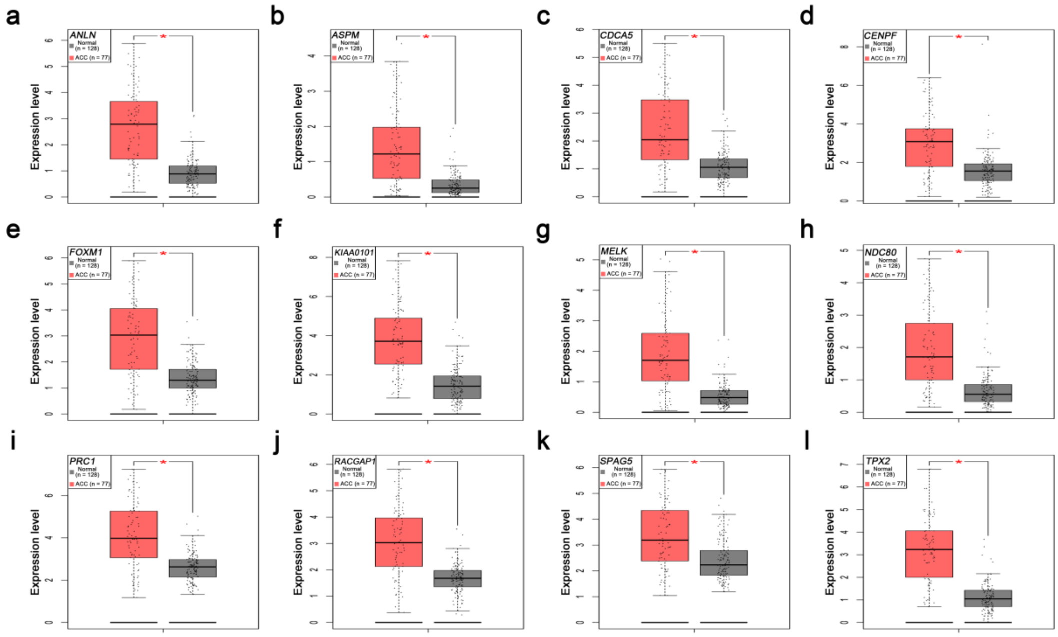

Supplementary Figure S4. Validation of the real hub genes using GEPIA (TCGA ACC). Validation of the gene expression levels of real hub genes between normal adrenal cortex and adrenocortical carcinoma samples (based on GEPIA database). (a) ANLN, (b) ASPM, (c) CDCA5, (d) CENPF, (e) FOXM1, (f) KIAA0101, (g) MELK, (h) NDC80, (i) PRC1, (j) RACGAP1, (k) SPAG5, (l) TPX2.
