# Supplementary material for: Co-expression Network Analysis of Biomarkers for Adrenocortical Carcinoma
Source: Front Genet. 2018 Aug 15;9:328. doi: 10.3389/fgene.2018.00328 (PMC6104177; doi:10.3389/fgene.2018.00328)
Supplement: Supplementary file 10 [file Image_5.pdf]

Supplementary Figure S5

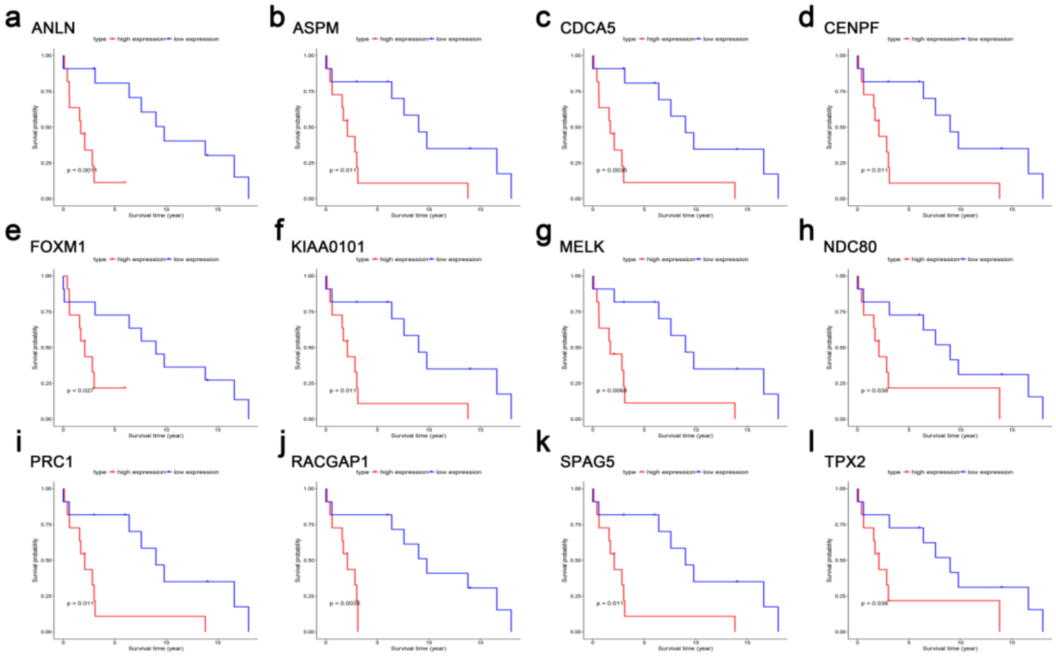

Supplementary Figure S5. Validation of the survival analysis using GSE19750. (a) ANLN, (b) ASPM, (c) CDCA5, (d) CENPF, (e) FOXM1, (f) KIAA0101, (g) MELK, (h) NDC80, (i) PRC1, (j) RACGAP1, (k) SPAG5, (l) TPX2.
