# Supplementary material for: Co-expression Network Analysis of Biomarkers for Adrenocortical Carcinoma
Source: Front Genet. 2018 Aug 15;9:328. doi: 10.3389/fgene.2018.00328 (PMC6104177; doi:10.3389/fgene.2018.00328)
Supplement: Supplementary file 11 [file Image_6.PDF]

Supplementary Figure S6

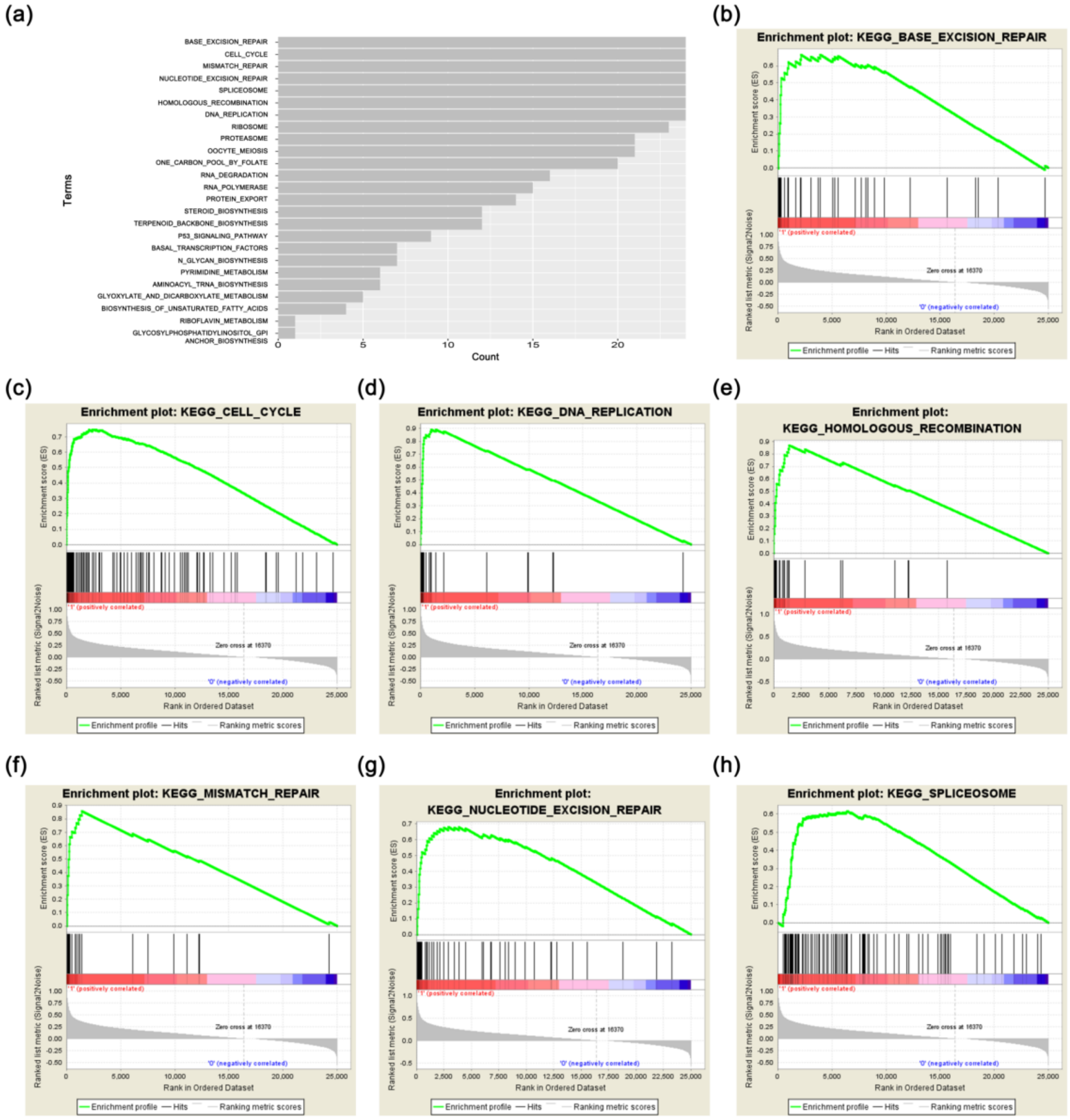

Supplementary Figure S6. Gene set enrichment analysis (GSEA). (a-b) “base excision repair”, (c) “cell cycle”, (d) “DNA replication”, (e) “homologous recombination”, (f) “mismatch repair”, (g) “nucleotide excision repair” and (h) “spliceosome”. Only the listed 7 common functional gene sets enriched in ACC samples with ANLN highly expressed.
